# Computerized cognitive training in people with depression: a protocol for a systematic review and meta-analysis

**DOI:** 10.1186/s13643-021-01872-6

**Published:** 2022-01-06

**Authors:** Amit Lampit, Nathalie H. Launder, Ruth Minkov, Alice Rollini, Christopher G. Davey, Carsten Finke, Nicola T. Lautenschlager, Hanna Malmberg Gavelin

**Affiliations:** 1 Department of Psychiatry, University of Melbourne, Parkville, Australia; 2 Department of Neurology, Charité – Universitätsmedizin Berlin, Berlin, Germany; 3 Berlin School of Mind and Brain, Humboldt-Universität zu Berlin, Berlin, Germany; 4 NorthWestern Mental Health, Melbourne Health, Melbourne, Victoria Australia; 5 Department of Psychology, Umeå University, Umeå, Sweden

**Keywords:** Depression, Major depressive disorder, Computerised cognitive training, Meta-analysis

## Abstract

**Background:**

People with depression often present with concurrent cognitive impairment. Computerized cognitive training (CCT) is a safe and efficacious strategy to maintain or enhance cognitive performance in a range of clinical populations. However, its efficacy in people with depression and how it varies across populations and design factors are currently unclear.

**Methods:**

We searched MEDLINE, EMBASE, and PsycINFO from inception to 13 July 2021 for randomised controlled trials examining the efficacy of CCT vs any control condition on cognitive, mood, psychiatric symptoms, psychosocial, and daily functioning in adults with depression. Eligible samples include studies specifically targeting people with major depressive disorder as well as those with other diagnoses where at least 50% of the sample meets the clinical criteria for depression, with the exception of major psychiatric disorders or dementia. The primary outcome is change in the overall cognitive performance. Multivariate analyses will be used to examine the effect sizes on each outcome category as well as possible effect modifiers and correlations between categories. The risk of bias will be assessed using the Cochrane risk of bias tool version 2.

**Discussion:**

To the best of our knowledge, this will be the first systematic review and meta-analysis of narrowly defined CCT across clinical populations with depression. We aim to investigate not only whether CCT is efficacious for cognition, but also how such effects vary across design factors, what other clinically relevant outcomes might respond to CCT, and the extent to which they differ across populations.

**Systematic review registration:**

PROSPERO CRD42020204209

**Supplementary Information:**

The online version contains supplementary material available at 10.1186/s13643-021-01872-6.

## Background

Cognitive impairment is a common feature of depression [1], affecting multiple cognitive domains not only in symptomatic but also remitted states [2]. Although prevalence estimates may vary across clinical settings and definitions, some studies suggest that 79–91% of people with depression may present objective impairment of ≥ 1 standard deviation in two or more cognitive domains [3], while subjective cognitive complaints may be present 85–94% and 39–44% of the time during depressive and remitted states, respectively [4]. Few trials of antidepressants and psychotherapy reported objective cognitive outcomes, and there is weak evidence that these interventions are efficacious for cognitive functioning when provided without explicit cognitive remediation [5–8].

Computerised cognitive training (CCT) is a key component of cognitive remediation and has received increasing interest for targeting cognitive and functional outcomes in depression and a range of other mental disorders [6, 9]. CCT is different from other psychological interventions by focusing on repeated and controlled practice on cognitively demanding tasks targeting one or more cognitive domains, as opposed to explicit learning of compensatory strategies [6, 9]. CCT is inherently safe, typically adaptive to individual needs, provides ongoing feedback and can be delivered inexpensively in a range of healthcare and community settings. Meta-analyses of randomised controlled trials (RCTs) investigating CCT by itself or in combination with other strategies have reported small-to-moderate effect sizes for not only cognition but also for psychosocial and functional outcomes in schizophrenia [10, 11], psychosis [12] and mild cognitive impairment [13]. However, effect size estimates are often heterogeneous and vary across populations and outcomes, as well as intervention design factors such as training content, dose, and supervision [10, 11, 14].

The efficacy of CCT in people with depression has been investigated in four systematic reviews with meta-analysis [15–18] encompassing a total number of 8 [18] to 21 [17] studies. All reviews found small to moderate effect sizes for global and domain-specific cognitive performance and three reported moderate effects sizes for depressive symptoms [15–18]. However, all four reviews combined RCTs with non-randomised as well as CCT with other approaches to cognitive remediation. Moreover, all but one review [15] used univariate analyses, which tend to underestimate heterogeneity when combining dependent effect sizes (such as multiple cognitive tests) and thus limit the investigation of potential effect moderators [19]. Finally, all four reviews were specific to populations with a primary diagnosis of major depression, thus excluding the body of evidence for CCT in depression comorbid to other disorders.

Therefore, while results of preliminary meta-analyses as well as those in other populations are encouraging, the potential of CCT as an effective intervention for cognition and function in people with depression has yet to be systematically and robustly evaluated. Moreover, investigations of the extent to which design factors such as population characteristics, intervention strategies, control comparisons, and study quality may relate to clinical outcomes are required in order to inform practice guidelines [6].

## Objectives

The aim of this review is to evaluate the efficacy of CCT on cognitive, mood, psychosocial and functional outcomes in adults with depression. Specifically, we aim to:

1. Investigate the efficacy of CCT on cognitive, mood, psychosocial outcomes, and daily functioning in comparison with active or passive control.
2. Examine the study and intervention design factors that could moderate CCT effects across studies in each domain
3. Evaluate the strength and quality of the evidence for CCT in depression
4. Suggest recommendations for future research and practice in the field

## Methods

This protocol adheres to the Preferred Reporting Items for Systematic Review and Meta-analysis Protocols (PRISMA-P) guidelines [20]. The PRISMA-P checklist is provided as Additional file 1.

### Eligibility criteria

Consistent with our previous systematic reviews of CCT [13, 14, 21], we will include studies that meet the following criteria.

#### Types of studies

RCTs studying the effects of CCT on one or more cognitive, mood, psychosocial, or functional outcome in people with depression. Eligible studies will provide neuropsychological testing or clinical outcome measures (e.g. depression scales) at baseline and post-CCT intervention. Non-randomised trials will be excluded. Unpublished RCTs or those published as conference abstracts, theses, or monographs will be eligible if data needed for the analysis and appraisal can be obtained from the authors.

#### Types of participants

Eligible participant groups will be relatively broad in order to ensure results are relevant across clinical settings as well as to examine whether the efficacy varies across populations, including those with other chronic disorders [22]. Therefore, included studies would have recruited adults (aged ≥ 18 years, including older adults) with depression at baseline, established according to standard diagnostic criteria (e.g. Research Diagnostic Criteria, DSM-5, ICD-10), diagnostic interviews, expert clinical diagnosis, or median score greater than a cutoff on an established clinical measure (e.g. BDI ≥ 14, GDS-30 ≥ 10), at any clinical stage. These may include, for example, samples of treatment-resistant or recurrent depression, those on chronic pharmacological treatment, in- or outpatients, or mixed samples. There will be no limitations for studies where some or all the sample uses concurrent antidepressant medications. Studies who sampled people from a broader clinical sample (e.g. mixed psychiatric samples, mild cognitive impairment, multiple sclerosis) meeting the criteria for depression will be included. However, studies recruiting solely from a psychiatric sample other than depression (e.g. all included patients with schizophrenia) will be excluded. In the case of mixed psychiatric samples, if ≥ 50% of the sample includes people with other major psychiatric disorders or receiving antipsychotic medication, the study will be included only if data for those without the concurrent disorder or medication use can be obtained from the report or authors. Similarly, if ≥ 50% of the sample includes people with dementia, the study will be included only if data for participants without dementia can be obtained as CCT is unlikely to be efficacious in dementia [13]. In all other cases, we will try to obtain separate data but will not categorically exclude the study if otherwise eligible. A clinical panel including a consultant psychiatrist (CGD), old age psychiatrist (NTL), neurologist (CF), and neuropsychologist (HMG) will review and approve the inclusion decisions.

#### Types of interventions

Minimum of 3 h [23] of practice on standardized computerized tasks or video games with clear cognitive rationale, administered on personal computers, mobile devices, or gaming consoles. Studies combining CCT with other non-pharmacological interventions (e.g. psychotherapy, physical exercise, brain stimulation) or with pharmacological interventions will be eligible as long as the CCT condition is the only key difference between the two groups. That is, studies will be included only if the contrast between the arms allows to delineate the effect of CCT from the composite intervention; thus, studies comparing, e.g., CCT + antidepressant to CCT + placebo will be excluded as such designs do not provide useful information regarding the effects of CCT.

#### Types of comparators

Eligible control conditions include wait-list, no-contact, and active (e.g. sham CCT, recreational activities) control groups. Alternative treatments (e.g. pharmacological, physical exercise) will be eligible if provided similarly to both groups. All eligible controls in multi-arm studies will be included.

#### Types of outcomes

One of the eligible outcomes is change in performance from baseline to post-intervention in non-trained measures of cognition (global or domain-specific), assessed through standardised neuropsychological tests or close equivalents (e.g. a computer-based version of a common neuropsychological test). Additional outcomes include validated measures of mood, psychiatric symptoms (e.g. anxiety, neuropsychiatric symptoms), subjective cognitive function, and daily functioning. Outcomes will be excluded if they were used as (or closely resemble) training tasks or if they were exploratory in nature (i.e. do not resemble common neuropsychological tests). In studies reporting more than one outcome measure per category, all eligible outcome measures will be included and pooled within studies (see the “Data synthesis” section). The primary outcome will be overall cognitive performance, defined as the mean effect size across all cognitive outcomes in a study [11, 13, 14, 21]. Secondary outcomes are domain-specific cognitive performance, classified according to the CHC-M framework [24], global cognition, subjective cognition, mood, other psychiatric symptoms, psychosocial functioning, and daily function.

### Search strategy

We searched MEDLINE, EMBASE, and PsycINFO through the OVID interface for eligible articles from inception to 13 July 2021. No restrictions on language or type of publication will be applied. The electronic search will be complemented by hand-searching the references of the included articles and previous reviews as well as clinical trial registries. The full search strategy is provided in Additional file 2.

### Study selection

Literature search results will be uploaded to a single Covidence library. Duplicates will be removed, and articles identified from other sources will be added. Initial screening for eligibility based on the titles and abstracts will be conducted by two independent reviewers. Full-text screening of potentially relevant articles will be conducted by two independent reviewers. Disagreements at each stage will be resolved by consensus or by the involvement of a senior reviewer (AL). The final list of included studies will be reviewed by at least two members of the clinical panel (CGD, NTL, CF, and HMG).

### Data extraction

Data will be extracted to a piloted Excel spreadsheet by one reviewer, and a senior reviewer (AL or HMG) will check data entry for each entry and cross-check with the original manuscript. Any disagreements will be resolved by consensus or by the involvement of a third reviewer if necessary. If any additional information is needed, we will contact the corresponding authors of the studies. The following data items will be extracted:

- Study information: first author, year of publication, and study location
- Population: mean age, per cent female, clinical characteristics at baseline (diagnostic criteria, mean depression scores, clinical stage), co-morbid disorders, medication use, mean MMSE score, or equivalent (older samples only)
- Intervention: type of CCT, programme used, training content, delivery format (supervised or unsupervised), total training duration (h), session frequency (sessions/week), session length (min), total number of sessions, intervention duration (weeks), and adjacent treatments
- Comparator: type of control and control group activity
- Outcome: name of measure, summary data for each group (e.g. mean, standard deviation, sample size) at baseline and post-intervention, and cognitive or clinical domain

Intention-to-treat data will be preferred if reported. Data will be extracted as means and standard deviation for each time point or change scores. If such information is not available, data in other formats (e.g. effect sizes and confidence intervals) will be used if the article provides sufficient information to reliably calculate the standardised mean difference. If these data are unavailable, the authors will be contacted to obtain missing data.

### Risk of bias assessment

The risk of bias in individual RCTs will be assessed using the revised Cochrane Risk of Bias tool (RoB 2) [25]. Low, high, or some concerns risk of bias will be determined for each of the following domains:

1. Bias arising from the randomization process
2. Bias due to deviations from intended interventions
3. Bias due to missing outcome data
4. Bias in measurement of the outcome
5. Bias in selection of the reported result
6. Overall bias

Studies with “some concerns” or “high” risk of bias in domains 3 or 4 will be considered as having some concerns or high risk of bias, respectively. Two independent reviewers will assess the risk of bias, and disagreements will be resolved by consensus or consulting a third reviewer if necessary.

### Data synthesis

Analyses will be conducted using the packages metafor, metaSEM, robumeta, and clubSandwich for R. Between-group differences in the change from baseline to post-intervention will be converted to standardized mean differences and calculated as Hedges’ *g* with 95% confidence interval for each eligible outcome measure. Pooling of outcomes across studies will be conducted using random-effects models. All eligible outcomes per analysis will be used, accounting for the dependency structure of effect sizes within studies [19, 26]. Sensitivity analyses for the primary outcome will be conducted by comparing the results from multilevel and robust variance estimation models. Analyses of secondary outcomes will be contingent on the availability of at least three studies for analysis.

Heterogeneity across studies will be quantified using tau^2^ and additionally expressed as a proportion of the overall observed variance using the *I*^2^ statistic [27, 28]. Prediction intervals will be calculated to assess the dispersion of effects across settings [29]. Provided sufficient statistical power for investigations of heterogeneity [30], potential moderators will be investigated using meta-regression models. Additional meta-regressions will examine the relationship between cognitive, mood, and functional effect sizes. If warranted, potential interactions across moderators will be tested on an exploratory basis using multivariate meta-regressions.

### Meta-bias(es)

The small study effect will be assessed by visually inspecting funnel plots of effect size vs standard error [31]. If at least 10 studies are available, the small study effect will be formally tested using a multivariate analogue of Egger’s test [32], i.e. a meta-regression using standard error as a covariate. Subgroup analysis of the primary outcome will be conducted based on the overall RoB 2 scores.

### Confidence in cumulative evidence

The strength of the evidence will be assessed and summarized qualitatively based on the risk of bias for individual studies, precision of the effect estimates, heterogeneity across studies (including prediction intervals), and evidence for small study effects, with additional sensitivity analyses conducted if warranted.

## Discussion

Depressive symptoms and their associated cognitive impairments are prevalent and heterogeneous. Our eligibility criteria allow for the inclusion of different presentations and definitions of depression in clinical practice, while including only RCTs of narrowly defined CCT. Combined with our multivariate analysis approach, these criteria will allow us to examine the clinical and intervention design factors as sources of heterogeneity and potential effect modifiers. As such, we aim to examine not only whether CCT is efficacious, but also for what outcomes and in whom, and intervention and study design elements appear to be most promising in future trials and clinical practice.

## Supplementary Information

**Additional file 1:** PRISMA-P checklist.

**Additional file 2:** Search strategy (MEDLINE, EMBASE and PsycINFO on Ovid).

## Data Availability

Not applicable.

## Abbreviations

BDI: Beck Depression Inventory
CCT: Computerised cognitive training
CHC-M: Cattell-Horn-Carroll-Miyake
DSM-5: Diagnostic and Statistical Manual of Mental Disorders, 5th Edition
GDS: Geriatric Depression Scale
ICD-10: International Statistical Classification of Diseases, 10th Revision
MMSE: Mini-Mental State Examination
RCT: Randomised controlled trial
