# Supplementary material for: Computerized cognitive training in people with depression: a protocol for a systematic review and meta-analysis
Source: Syst Rev. 2022 Jan 6;11:6. doi: 10.1186/s13643-021-01872-6 (PMC8740357; doi:10.1186/s13643-021-01872-6)
Supplement: Supplementary file 2 — Additional file 2: Search strategy (MEDLINE, EMBASE and PsycINFO on Ovid). [file 13643_2021_1872_MOESM2_ESM.docx]

**Additional file 2.** Search strategy (MEDLINE, EMBASE and PsycINFO on Ovid)

| # 1 | ((cognit* or attention or neurocognit* or neuropsycholog* or memory or reasoning or executive) adj2 (training* or rehabilitat* or remediat* or stimulat* or exercis* or retrain*)).mp. |
| --- | --- |
| # 2 | ((brain) adj2 (training* or rehabilitat* or remediat* or retrain*)).mp. |
| # 3 | (speed adj3 training).mp |
| # 4 | 1 or 2 or 3 |
| # 5 | Exp Depressive Disorder/ |
| # 6 | Depression/ |
| # 7 | Depress*.tw,kw |
| # 8 | 5 or 6 or 7 |
| # 10 | 4 and 8 |
